# A cost-effectiveness analysis of iStent *inject* combined with phacoemulsification cataract surgery in patients with mild-to-moderate open-angle glaucoma in France

**DOI:** 10.1371/journal.pone.0252130

**Published:** 2021-06-10

**Authors:** Kaspar Nieland, Antoine Labbé, Cedric Schweitzer, Gaetan Gicquel, Joris Kleintjens, Amrita Ostawal, Maarten Treur, Heather Falvey

**Affiliations:** 1 Pharmerit International, Rotterdam, The Netherlands; 2 Department of Ophthalmology III, Quinze-Vingts National Ophthalmology Hospital, IHU FOReSIGHT, INSERM-DHOS CIC 1423, Paris, France; 3 Department of Ophthalmology, Ambroise Paré Hospital, AP-HP, Paris Saclay University, Boulogne-Billancourt, France; 4 CHU Bordeaux, Department of Ophthalmology, Univ. Bordeaux, ISPED, INSERM, U1219 – Bordeaux Population Health Research Centre, Bordeaux, France; 5 Glaukos Corporation, Paris, France; 6 Pharmerit International, Berlin, Germany; 7 Glaukos Corp, San Clemente, California, United States of America; LV Prasad Eye Institute, INDIA

## Abstract

**Objective:**

To investigate the cost-effectiveness of implementing iStent *inject* trabecular bypass stent (TBS) in conjunction with cataract surgery (Cat Sx) in patients with mild-to-moderate glaucoma from a societal perspective in France. The secondary objective was to explore the economic impact of iStent *inject* TBS in patients who comply to different degrees with their anti-glaucoma medications.

**Methods:**

A previously published Markov model was adapted to estimate the cost-effectiveness of treatment with iStent *inject* TBS + Cat Sx versus Cat Sx alone over a lifetime time horizon in patients with mild-to-moderate open-angle glaucoma in France. Progression was modeled by health states reflecting increasing stages of vision loss. Disease progression was obtained from the two-year randomized clinical trial assessing safety and effectiveness of both interventions. French specific health-state utilities and costs were obtained through a targeted literature review. Model structure and inputs were validated by French ophthalmologists. Outcomes were expressed as incremental cost per quality-adjusted life-year (QALY) gained. The robustness of results was tested through sensitivity analyses.

**Results:**

iStent *inject* TBS + Cat Sx reduced the number of medications needed and risk of blindness. Incremental cost and QALYs were €75 and 0.065 leading to an incremental cost-effectiveness ratio (ICER) of €1,154/QALY gained. ICER ranged from dominating for non-persistent patients to €31,127 patients fully persistent with their medication regime. Results from one-way sensitivity analysis had a maximum ICER of €29,000 when varying input parameters. iStent *inject* TBS + Cat Sx had an 86% chance of being cost-effective at a willingness-to-pay threshold of €30,000 per QALY gained.

**Conclusion:**

Results demonstrate that iStent *inject* TBS + Cat Sx is a cost-effective intervention for intraocular pressure reduction when compared to Cat Sx alone in France.

## Introduction

Glaucoma is a neurodegenerative disease that is characterized by progressive, and largely asymptomatic vision loss caused by optic nerve damage, and is the second leading cause of irreversible blindness worldwide [1–4]. A population-based study of the elderly in France found that 7.5% of 82-year-old had glaucoma, of which 50% had progressed to moderate glaucoma, and 34.2% to advanced glaucoma [5]. Glaucoma is commonly treated with medication, selective laser trabeculoplasty (SLT) and filtering surgery according to the European Glaucoma Society (EGS) guidelines, however, these treatments have their limitations. First, most patients are not taking their medications correctly, and issues with compliance and persistence are thought to be one of the main barriers to effective glaucoma treatment [6, 7]. A retrospective study in France revealed that only 45% of patients are persistent to first-line therapy with medications after one year [8]. Poor persistence can be due to the asymptomatic symptoms of glaucoma, which makes the patient not realize the importance of medication. Another reason for poor persistence is that medication can cause frequent adverse reactions like corneal erosion and superficial punctate keratitis, and also aggravate the ocular surface disease (OSD) that often coexists with glaucoma [9–11]. Second, studies have shown that response to SLT, defined as 20% reduction in intraocular pressure (IOP) after 6–12 months, is achieved in only 55–82% of all cases, and SLT is associated with adverse effects like postoperative inflammation [12, 13]. Last, filtering surgery, although effective, is only recommended when other forms of therapy have failed, due to the invasive nature of filtering surgery and the associated long-term risks such as the development of persistent corneal edema and dysesthesia [14, 15].

Glaucoma frequently coexists with cataract, as the prevalence of both diseases increases with age, and thus the patient will commonly need treatment for both [16]. Cataract surgery (Cat Sx) alone may result in a reduction in ocular pressure, but is often not sufficient enough to control IOP and may need to be combined with filtering surgeries with its associated risks [17, 18]. As a consequence, there remains a need for alternative treatment options for patients with coexisting glaucoma and cataract. The iStent *inject* trabecular bypass stent (TBS) is a procedure that effectively reduces IOP and increases outflow facility, and can be implanted during Cat Sx [19]. The device was extensively studied in a randomized clinical trial (RCT) comparing 2-year data post-surgery of iStent *inject* TBS + Cat Sx (N = 380) vs Cat Sx alone (N = 118) in patients with mild-to-moderate OAG. At 24 months, 75.8% of treatment eyes versus 61.9% of control eyes experienced ≥20% reduction from baseline unmedicated diurnal IOP, and 84% of eyes treated with iStent *inject* versus 67% of control eyes were not treated with ocular hypotensive medications at 23 months. The safety profile of iStent *inject* was favorable and the overall rate of adverse events was comparable between both treatment arms [20]. Moreover, iStent *inject* TBS requires minimal additional time from the surgeon as it can be inserted through a small corneal incision during Cat Sx. This makes it a suitable treatment option for patients with mild- to moderate glaucoma undergoing Cat Sx. iStent *inject* TBS + Cat Sx has been reviewed and approved by the Haute Autorité de Santé with a grade III amélioration du service attendu (ASA).

Understanding not only the clinical value of iStent *inject* TBS but also its cost-effectiveness is important for an investment decision by a healthcare provider with limited resources. In this study we therefore investigated the cost-effectiveness of implementing the iStent *inject* TBS in conjunction with Cat Sx in patients with mild-to-moderate glaucoma from a societal perspective in France. Moreover, we explored the cost-effectiveness of iStent *inject* TBS in patients who to different degrees comply with their anti-glaucoma treatment.

## Methods

A cost-effectiveness analysis (CEA) is a form of economic evaluation where both the costs and consequences of treatments or health programs are examined [21]. These analyses are incorporated into reimbursement decision-making. The typical outcome of a CEA is the incremental cost-effectiveness ratio (ICER), expressed as incremental cost per quality-adjusted life-year (QALY) gained [22].

### Model structure

A previously published Markov model [23] was adapted in accordance with French health-economic guidelines [24, 25] to estimate the economic impact of iStent *inject* TBS in conjunction with Cat Sx in France. A Markov model is a mathematical method for estimating costs and health consequences for patients with a disease over time, and are useful for comparing various treatment alternatives for chronic diseases. Markov models typically describe a disease by discrete health states of varying degrees of severity [23].

**Fig 1:**
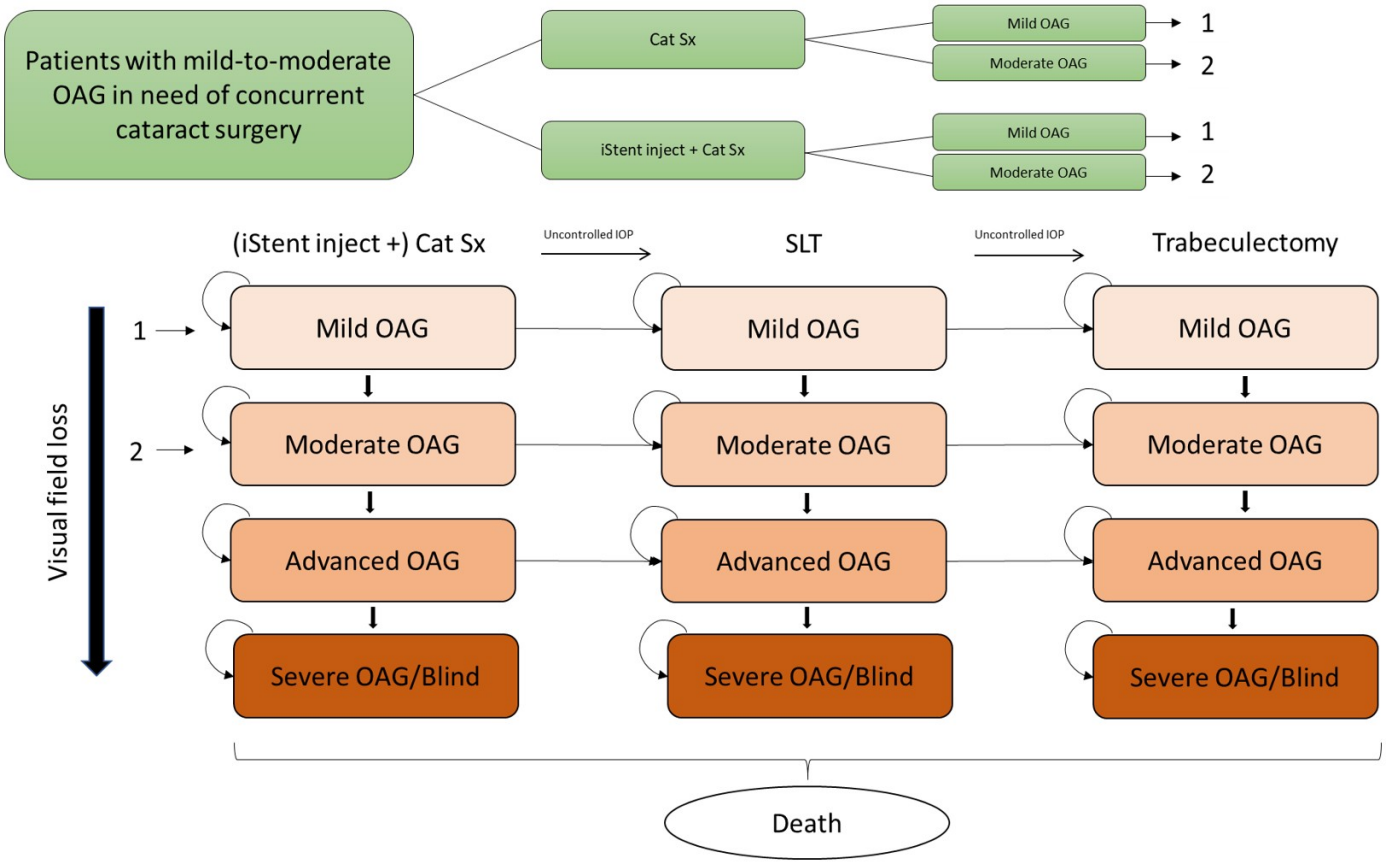
Model structure.

Our Markov model (Fig 1) reflects disease and treatment progression. Disease progression is defined by four health states that follow the natural progression of glaucoma according to an adapted Hodapp-Parrish-Anderson scale: (a) Mild OAG (0 to -6 decibel [dB]), (b) Moderate OAG (-6.01 to -12 dB), (c) Advanced OAG (-12.01 to -20 dB), (d) Severe OAG/blind (<-20 dB)., supplemented with (e) Death [26]. The pace of disease progression was based on the no-treatment arm of the Early Manifest Glaucoma Trial (EMGT) of newly diagnosed treatment-naïve OAG patients [27]. Corresponding effect of Cat sx ± iStent *inject* TBS on deceleration of disease progression were obtained from the two-year randomized clinical trial assessing the post-operative safety and effectiveness of both interventions in patients with mild-to-moderate OAG [20].

Patients had mild or moderate OAG and were treated with iStent *inject* TBS + Cat Sx or Cat Sx alone upon model entry. Subsequently, the glaucoma patients were treated with medication, SLT and filtering surgery to lower IOP and slow down disease progression. The probability of receiving subsequent treatments was based on the observed risk of visual field (VF) defect and optic disc damage in EMGT [27].

The OAG patient’s lifetime was simulated and costs, QALYs and blind eyes per patient were collected. Key model characteristics are summarized in Table 1. Further details on the model structure and assumptions used in the model have been described in a previous publication [23].

**Table 1:** Key model characteristics.

Elements	Description
Model design	Markov model
Health states	Glaucoma severity: Mild, moderate, advanced and severe/blind Absorbing state: Death
Perspective	Societal perspective
Target patient population	Mild-to-moderate OAG patients
Treatment intervention and comparators	In patients in need of cataract surgery: TBS with cataract surgery vs. cataract surgery alone
Subsequent treatments	First subsequent treatment is 360 SLT, second subsequent treatment is filtering surgery
Time horizon	Lifetime
Markov cycle	1 month
Discount rate	4%
Cost Data Included	Costs for surgery, medical device, medications, filtering surgery, SLT, ophthalmologist and hospital practitioner visits, routine IOP and VF defect tests, adverse events, transportation and disability benefits
Mean time to medication discontinuation	32.2 months
Year of Cost & Currency	2019; Euros €
Analyses	Probabilistic base case analysis, one-way sensitivity analyses and scenarios

IOP = intraocular pressure, OAG = open-angle glaucoma, SLT = trabeculoplasty, TBS = trabecular bypass stent, VF = visual field.

### Model input data

A targeted literature review for French-specific inputs was conducted. The model structure, treatment pathway, assumptions and HCRU were validated by French ophthalmologists.

#### Model population

The baseline patient population was 65-year old with mild-to-moderate OAG, with a mean IOP of 24.8 mmHg and on 1.6 medications. The mean age at treatment initiation in France was not identified in the literature. Instead, an Italian study was used to estimate the mean age of the target population, under the assumption that the onset of disease and disease progression of glaucoma is relatively similar between Italy and France [28]. In absence of French specific data, baseline distribution of glaucoma severity at treatment initiation from a large US claims database was used, with 83.1% mild and 16.9% moderate glaucoma [29]. Mean unmedicated IOP at baseline and mean number of medications was obtained from the RCT that evaluated the efficacy and safety of iStent *inject* TBS which has been described earlier [20].

#### Disease progression and adverse events

Details on how disease progression was modeled can be found in Patel et al. (2019) [23]. Adverse events with a difference of at least 3% between iStent *inject* TBS + Cat Sx and Cat Sx alone in the RCT were included in the model, which were stent obstruction (6.2% vs 0%) and hyperemia (0.8% vs 5.9%) [20].

#### Medication persistence

Medication discontinuation was derived from Belhassen et al. (2016) [8], reporting medication persistence in glaucoma patients in France over a 12 month follow-up period for four drug classes: prostaglandins, beta-blockers, beta-blocker-combination therapies, and carbonic anhydrase inhibitors. The 1-year data was extrapolated and adjusted for their relevant French market shares [30].

#### Health care resource utilization (HCRU), costs and utilities

French tariffs are reported in 2019 Euros (€) and costs extracted from the literature were inflated to 2019 values using the French consumer price index [31].

#### Health care resource utilization (HCRU)

Mean annual number of ophthalmologist consultations, hospital practitioner consultations, VF defect tests, optic disc imaging and proportion of surgeries taking place in public and private hospitals was elicited from French ophthalmologists. Twenty-five percent of surgeries were assumed to take place in public hospitals and 75% in private hospitals. HCRU details can be found in the online appendix.

#### Surgery, medication and adverse event costs

Surgery costs were obtained from French diagnostic related groups (DRG) for public and private hospitals [32]. The estimated average cost across private and public hospitals was €1,427 for iStent *inject* TBS + Cat Sx, €1,083 for Cat Sx alone, €125 for SLT and €1,367 for filtering surgery. Costs for ophthalmologists and hospital practitioners consultations were €36 [33].

Medication costs were obtained from the French claims database of MedicAm [30]. A shelf life of 1 month, and 21% wastage for all medication bottles was used and validated by French ophthalmologists [34]. The weighted average medication cost per month including 21% wastage and a dispensing fee of €1.02 of which 65% is reimbursed was estimated to be €9.31 [35]. If there was an option between several brands or sizes for one active ingredient, the most economical option was used.

#### Direct non-medical costs

Disability benefits for blind patients and transportation costs were included. For blind patients, a disability benefit of €900 per month was applied [36]. An ophthalmology visit was associated with a transportation cost of €51 [37].

#### Utilities

Health state and adverse event specific utility values (a measure of quality of life) were elicited from the original Canadian model by Patel et al. (2019) [23], in absence of French specific utility data. The utilities were derived from a Dutch cross sectional survey of patients with OAG [38]. Health preference was measured by Health Utilities Index 3 using tariffs for the Canadian population.

### Outcomes and analyses

#### Base case analysis

A discount rate of 4% was applied to future costs and effects as per French health-economic guidelines [24, 25]. The willingness-to-pay (WTP) threshold used by National Institute for Health and Care Excellence (NICE) of €30,000 per QALY was used as a reference as no WTP threshold has been defined for France [39]. A WTP threshold represents the maximum the healthcare provider is prepared to pay for each additional QALY for their patients [40].

#### Sensitivity analyses

Sensitivity analyses were conducted to test the impact of the model assumptions on the outcomes. A One-way sensitivity analysis (OWSA) and probabilistic sensitivity analysis (PSA) was conducted to evaluate the effect of the uncertainty in the model. In the OWSA, the impact of extreme, yet plausible values of each model parameter on the ICER was investigated. In the PSA, samples were simultaneously drawn at random from the assigned probability distributions of the point estimate of all model inputs 1,000 times to generate an empirical distribution of patient outcomes. The outcomes of the PSA were used to estimate the probability of iStent *inject* TBS + CAT Sx being cost-effective at different willingness-to-pay (WTP) thresholds and visualized through a cost-effectiveness acceptability curve (CEAC).

#### Scenario analyses

Six scenarios were explored to test the impact of various assumptions and settings. First, we considered the effect of iStent *inject* TBS on patients non-compliant with their glaucoma medication. Second, we ran a scenario where we assumed all patients fully complied to their prescribed medication. Third, we used the mean time to medication discontinuation of 6.6 months as observed in Belhassen et al. (2016) [8] without extrapolating the data. Fourth, we investigated the effect of increasing the annual cost of blindness to €15,000, which was assumed a reasonable estimate of the aggregated average additional costs for blindness based on a study on cost of glaucoma-related blindness in Europe [41]. Finally, we tested two scenarios; one where the entire target population had mild disease, and one where the entire target population had moderate disease.

## Results

### Base case analysis

The results of the cost-effectiveness model showed that iStent *inject* TBS + Cat Sx treatment slows down disease progression to severe glaucoma by over half a year compared to Cat Sx alone. Quality-adjusted life expectancy in patients treated with iStent *inject* TBS + Cat Sx amounted to 11,05 QALYs versus 10,98 QALYs for Cat Sx alone. The corresponding total costs per patient were €13,949 and €13,874 respectively. The number of blind eyes per patient was 0.079 and 0.089 in the iStent *inject* TBS + Cat Sx versus Cat Sx patients. The reduction in blind eyes and slower disease progression for patients treated with iStent *inject* TBS + Cat Sx resulted in an additional 0.065 QALYs, and a reduction of 0.010 blind eyes at an additional total cost of €75 per treated patient compared to Cat Sx alone. The cost difference indicates that €984 of the €1,059 investment in iStent *inject* TBS is offset by reduction in costs elsewhere, such as fewer ophthalmologist visits, lower number of medications, fewer secondary surgeries and lower need for disability benefits. The outcomes of the base case analysis and scenarios are displayed in Table 2. The ICER of €1,154 per QALY gained is below the estimated acceptable WTP thresholds of €30,000 per QALY gained.

**Table 2:** Base case and scenario outcomes.

Parameter	TBS + Cat Sx	Cat Sx	Increment
**Deterministic Base Case**			
QALYs	11.05	10.98	0.065
Cost	€13,949	€13,874	€75
ICER			€1,154
**Scenarios**	**Incremental QALYs**	**Incremental costs**	**ICER**
Non-compliant patient	0.105	-€607	Dominates
Fully compliant patient	0.029	€888	€31,127
Only observed compliance	0,093	-€400	Dominates
Increase the annual cost of blindness to €15,000	0.065	-€377	Dominates
Mild disease only when receiving surgery	0.068	€162	€2,385
Moderate disease only receiving surgery	0.051	-€408	Dominates
TBS as a standalone procedure vs medication	0.172	-€167	Dominates

Cat Sx = cataract surgery, ICER = incremental cost-effectiveness ratio, QALY = quality-adjusted life-year, TBS = trabecular bypass stent.

The ICER of €1,154 per QALY gained is below the estimated acceptable WTP thresholds of €30,000 per QALY gained.

### Sensitivity analyses

**Fig 2:**
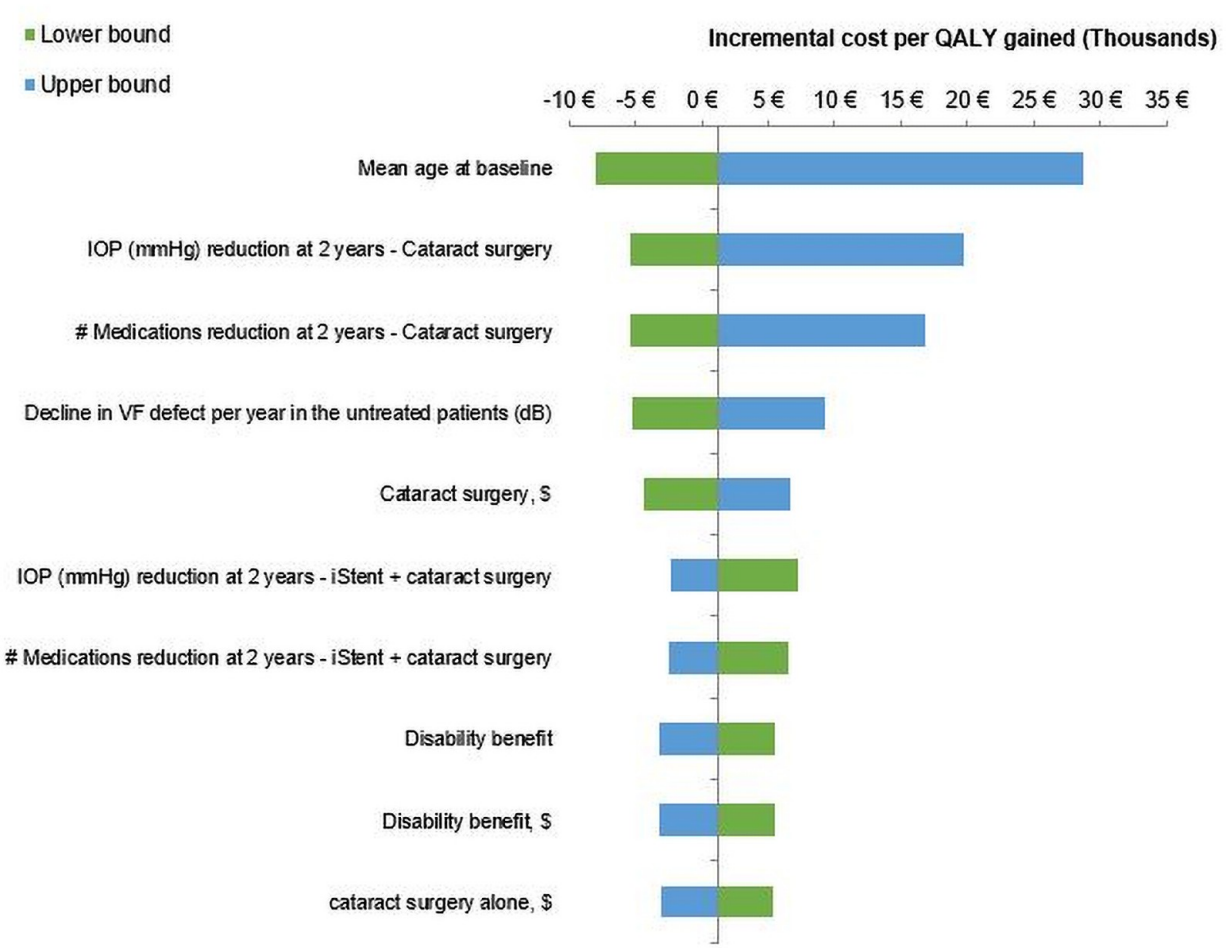
One-way sensitivity analysis.

The outcomes from the OWSA is presented as a tornado diagram (Fig 2) and displays the 10 input parameters with the largest impact on the ICER. The OWSA showed that the cost-effectiveness of iStent + Cat Sx is most sensitive to the age of the patient upon treatment initiation, as well as IOP reduction and medication reduction at 2 years in the Cat Sx arm. The ICER remained below €29,000 for all input parameters varied in the OWSA.

**Fig 3:**
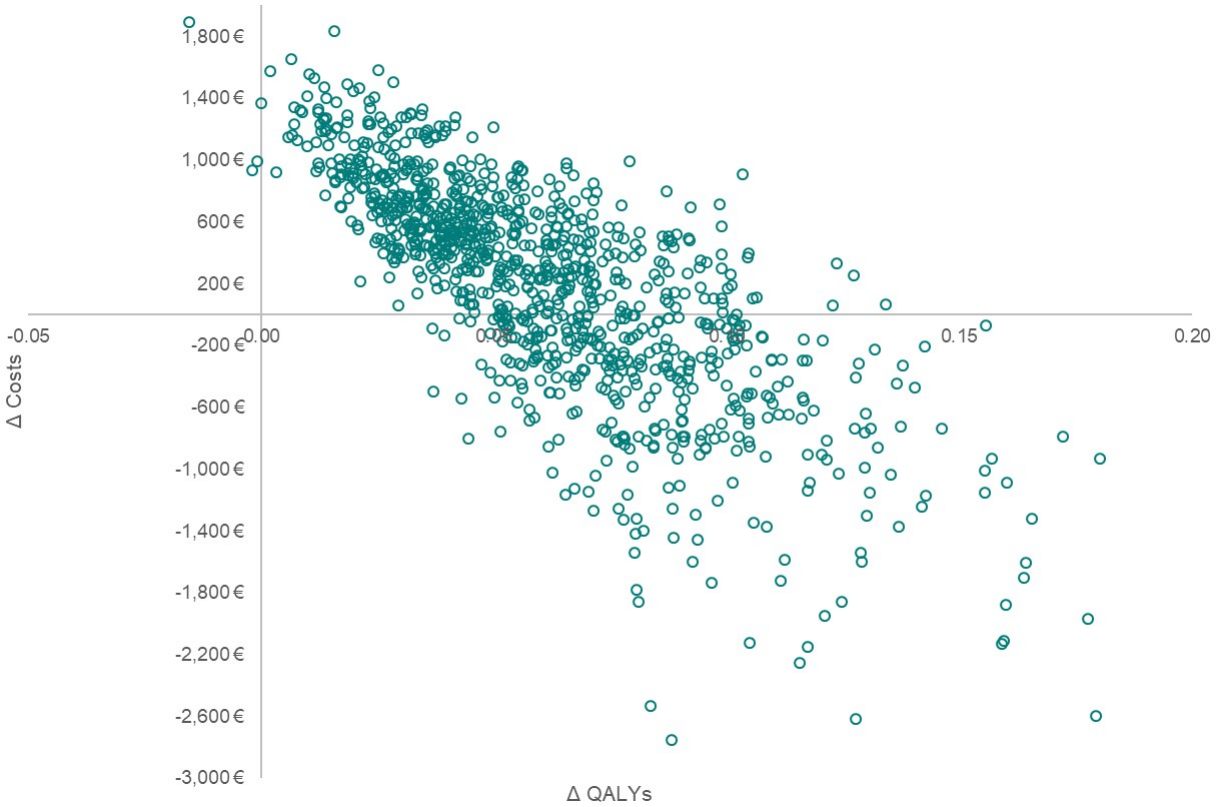
Scatterplot from probabilistic sensitivity analysis.

**Fig 4:**
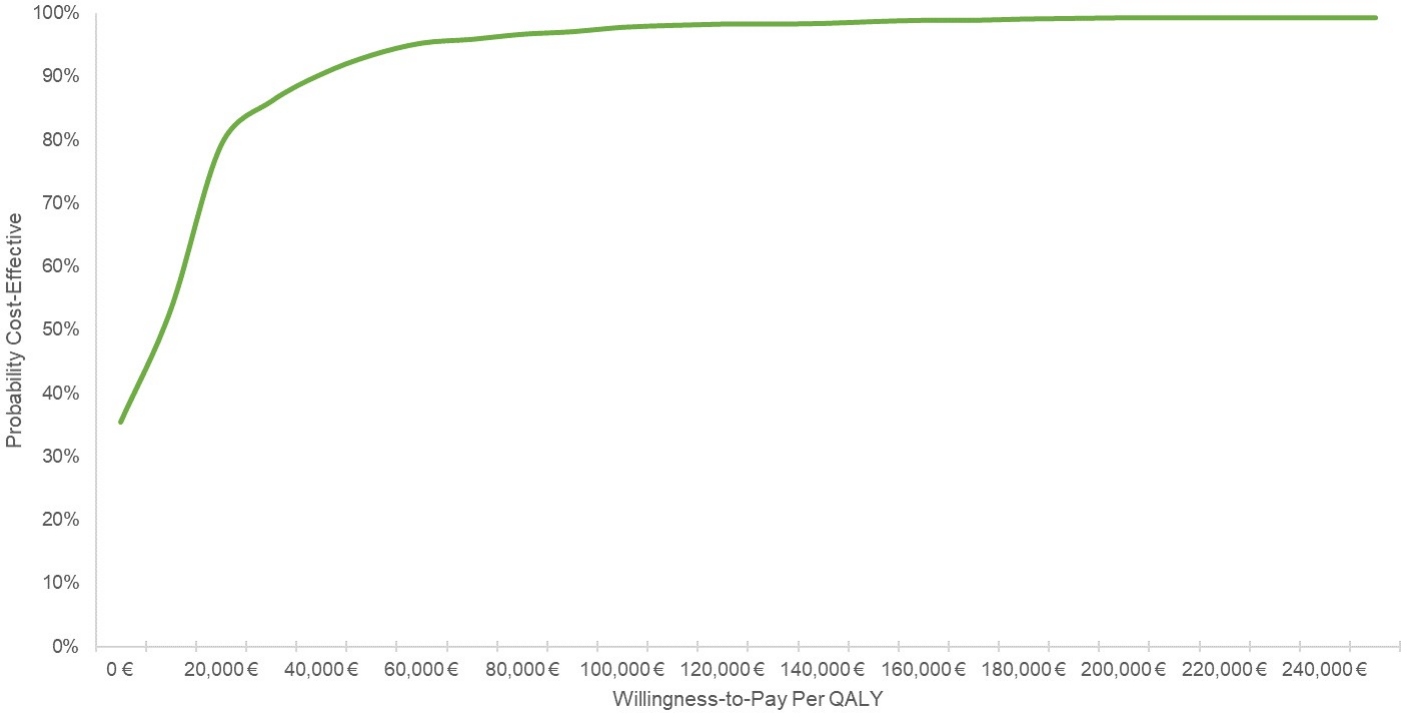
Cost-effectiveness acceptability curve.

Fig 3 shows the scatter plot based on the PSA. Fig 4 shows that at a WTP threshold of €30,000 per QALY gained, the probability that iStent *inject* TBS + Cat Sx will be cost-effective is 86% compared to Cat Sx alone.

### Scenario analyses

The scenarios showed that the patients’ persistence to their prescribed medication following surgery had a substantial impact on the outcomes of the analysis. The incremental QALY gain ranged from 0.029 for fully persistent patients to 0.105 for non-persistent patients, and the associated ICER ranged from €31,127 to dominating, meaning that iStent *inject* TBS + Cat Sx produced more QALYs at lower costs relative to Cat Sx alone.

In the scenario where the annual cost of blindness was set to €15,000, iStent *inject* TBS + Cat Sx was estimated to be the dominating strategy due to the larger proportion of patients losing vision in the Cat Sx alone population.

Finally, the ICERs when treating patients with only mild or only moderate disease was €2,385 and dominating respectively. This suggests that treating patients with mild OAG will grant the best quality-of-life improvements as their OAG is less likely to become severe. If patients are treated with moderate OAG, they are more likely to advance to severe OAG, where the main cost savings are achieved, but at the cost of reduced quality-of-life.

## Discussion

In our study, we examined the cost-effectiveness of iStent *inject* TBS + Cat Sx in French patients with mild-to-moderate OAG. The outcomes of the analyses showed that iStent *inject* TBS + Cat Sx maintains better IOP control and reduces the number of medications required over the patient’s lifetime compared to Cat Sx alone, increasing the patient’s quality of life. The results of our study suggest that iStent *inject* TBS + Cat Sx is a cost-effective treatment option for mild-to-moderate OAG patients in France with an 86% probability of being cost-effective at a WTP threshold of €30,000. The sensitivity analyses demonstrated that the age and medication persistence of the patient receiving surgery is a key driver for the cost-effectiveness.

The results of our study were in the same range as results from previous cost-effectiveness studies in Canada and Columbia. Patel et al. (2019) [23] reported that iStent TBS as a standalone procedure dominated a comparator strategy of medication alone from the Canadian public payer perspective. Similarly, in a study conducted by Ordóñez et al. (2019) [42], was iStent TBS found to be a highly cost-saving strategy in Colombia when compared to SLT.

For this study, we made a conservative estimate of the economic value of iStent *inject* TBS + Cat Sx. First, not all societal savings were included in the model. Further cost savings can be expected if costs associated with reduced needs for vision aids, early admission to a nursing home due to poor vision or blindness, and other informal care costs to society for assisting people with glaucoma were considered. In our model, the additional costs per year due to blindness was €10,800, but in 2005 these costs were reported to be between €12,000 and €19,000 annually [41]. When we explored this through a scenario, iStent *inject* TBS + Cat Sx was the dominant strategy compared to Cat Sx alone. Second, the reduction of medication use is an important observation, as patients with iStent *inject* TBS to a large degree can control their IOP without medication. In the clinical study, 84% of the iStent *inject* TBS-treated eyes meeting the study endpoint were not getting any ocular hypotensive medications after 2 years, compared to 67% in the control eyes [20], which is promising in a glaucoma population with known compliance and persistence issues. The observed medication persistence in Belhassen et al. (2016) [8] of only 45% 1 year after initiation can also be seen in other studies. Reardon et al. (2011) [43] did a systematic review of compliance and persistence among patients treated for glaucoma and ocular hypertension and found that only 56% of the days in the first therapy year could be dosed with the dispensed medication over 1 year of therapy, and only 31% of patients had not discontinued after 1 year. It is recommended to further explore the cost-effectiveness once long-term follow-up data of iStent *inject* TBS is available to fully understand the long-term benefits.

There are four main limitations of our cost-effectiveness study. First, due to lack of data in the public domain for France, an Italian study was used to estimate the mean age for the mild-to-moderate glaucoma patients, a US study was used to estimate the distribution of mild and moderate disease at baseline [29], and a survey on Dutch patients with Canadian tariffs was used to quantify the utility values [23, 44, 45]. These inputs may not be fully compatible to the French population, but were all thoroughly explored through various sensitivity and scenario analyses. Age was an important driver of the ICER and should be taken into account when considering iStent *inject* TBS, as the investment into iStent *inject* TBS is upfront, but the benefits accumulate over time. Although the benefit may be greater in younger patients, treatment to those in the upper bound of age would also be considered cost effective if considering a WTP threshold of €30,000. Second, medication persistence was extrapolated from a one-year observational study, which only gives us a rough estimate of a patient’s mean time-to-discontinuation. However, persistence scenarios were extensively explored, and iStent *inject* TBS + Cat Sx remained cost-effective even in a population that fully complied with their medication, although the benefits were significantly greater in non-persistent patients. Third, the efficacy data of TBS + Cat Sx and Cat Sx alone were extrapolated for a lifetime from the 2-year results of the RCT. Extrapolations are always associated with a degree of uncertainty that should be kept in mind when interpreting the results of the analysis. It is recommended updating this cost-effectiveness study once long-term data is available. Finally, only a limited societal perspective was used in the base case, so productivity loss and other costs like additional aid for blind people other than disability benefit is not included. The impact of these limitations was thoroughly tested through sensitivity and scenario analyses.

## Conclusion

In conclusion, iStent *inject* TBS offers a mechanism for IOP reduction that is effective and reduces the need for medications. iStent *inject* TBS implantation in conjunction with Cat Sx can be considered cost-effective in patients with mild-to-moderate OAG by improving the patient’s quality-of-life at very low incremental costs when compared to Cat Sx alone in France. The ICER remained below €30,000 when varying the efficacy, resource use, unit costs, natural disease progression, and glaucoma severity. Our study demonstrates that the iStent *inject* TBS devices provide a valuable alternative to patients with mild-to-moderate OAG in need of Cat Sx in France.
